# Protocol Dependence of Sequencing-Based Gene Expression Measurements

**DOI:** 10.1371/journal.pone.0019287

**Published:** 2011-05-06

**Authors:** Tal Raz, Philipp Kapranov, Doron Lipson, Stan Letovsky, Patrice M. Milos, John F. Thompson

**Affiliations:** Applications, Methods, and Collaborations Group, Helicos BioSciences, Cambridge, Massachusetts, United States of America; The University of Maryland, United States of America

## Abstract

RNA Seq provides unparalleled levels of information about the transcriptome including precise expression levels over a wide dynamic range. It is essential to understand how technical variation impacts the quality and interpretability of results, how potential errors could be introduced by the protocol, how the source of RNA affects transcript detection, and how all of these variations can impact the conclusions drawn. Multiple human RNA samples were used to assess RNA fragmentation, RNA fractionation, cDNA synthesis, and single versus multiple tag counting. Though protocols employing polyA RNA selection generate the highest number of non-ribosomal reads and the most precise measurements for coding transcripts, such protocols were found to detect only a fraction of the non-ribosomal RNA in human cells. PolyA RNA excludes thousands of annotated and even more unannotated transcripts, resulting in an incomplete view of the transcriptome. Ribosomal-depleted RNA provides a more cost-effective method for generating complete transcriptome coverage. Expression measurements using single tag counting provided advantages for assessing gene expression and for detecting short RNAs relative to multi-read protocols. Detection of short RNAs was also hampered by RNA fragmentation. Thus, this work will help researchers choose from among a range of options when analyzing gene expression, each with its own advantages and disadvantages.

## Introduction

Interest in quantifying levels of gene expression has been high ever since the first methods were developed to assess RNA differences across cell types and growth conditions. The methods and technologies have progressively improved to allow better sensitivity and inclusion of an ever greater number of transcripts in each assay. In the recent past, various forms of expression arrays have been the stalwart of gene expression analysis, but this technology is now rivaled by a more accurate, sensitive and versatile technology, RNA Seq (reviewed by [1], [2], [3], [4], [5]). RNA Seq provides a digital measure of RNA abundance represented by the sequence read counts in a region of interest as opposed to an indirect, analog signal from microarrays. In addition, it has a broader dynamic range, and is not dependent on having pre-existing knowledge about the transcriptome under study. Expression results from RNA Seq [6], [7], [8], [9], [10] and the related technologies of Digital Gene Expression (DGE) [11], [12], SageSeq [13], [14], CAGESeq [15], and PET Seq [16], which count 5′ and/or 3′ tags, have been compared to both microarrays and qPCR experiments and shown to produce highly accurate and reproducible results based on known spikes and other quality assessments. In addition to analysis of gene expression levels, RNA Seq is also able to discover novel transcripts, SNPs, splice junctions, and fusion transcripts as well as provide allele specific gene expression [17], [18], [19]. Additionally, paired read methods have been used in an attempt to maximize information about splicing and other long-range phenomena in RNA Seq experiments, but the ligation and amplification steps in such methods can introduce errors as evidenced by a relatively large proportion of paired reads appearing as chimeras arising from distinct genes (5–9%, [20]), most of which are artifactual.

Extensive efforts to characterize the reproducibility of microarray methodologies and platforms have been carried out previously [21], [22], showing that careful attention to methods can yield predictable reproducibility. The much greater dynamic range of RNA Seq and reduced susceptibility to artifacts caused by array cross-hybridization mean that RNA Seq data should have much higher reproducibility than hybridization-based approaches. While the ability of RNA Seq to detect all RNAs in a sample is an advantage with respect to identifying novel species and the complete range of transcripts within a cell, it is a disadvantage when total RNA is examined because such a high proportion of cellular RNA arises from ribosomal and mitochondrial sources. This limits the number of reads from other RNAs and thus the number of different transcripts that can be detected and the accuracy of their expression level. Hence, methods such as polyA RNA selection and ribosomal RNA depletion have been developed to minimize this problem. However, these fractionations have the potential to skew the RNA population that is detected. Similarly, the improved nature of the detection technology make attention to sample preparation techniques all the more critical as not only sample fractionation but also any sample manipulation including ligations, amplifications, and sample fragmentation can have an effect on the results observed. Understanding the impact that technical choices have on experimental outcome is critical if one is to properly evaluate expression profiles generated either within the same laboratory or across laboratories. Studies that fully characterize the benefits and limitations of RNA Seq technology have been initiated [23] but more comprehensive testing is still required. Our studies are intended to further explore what differences are likely to occur as protocols are varied.

We have performed multiple RNA Seq experiments and technical replications to assess protocol variations and reproducibility. The same samples have also been used for DGE expression profiles in order to compare single and multi-tag approaches for their impact on expression measurements. In this work, we will focus on gene expression and means for optimizing the accuracy and precision of those measurements for all cellular transcripts. The Helicos single-molecule sequencing technology is used because of its reduced biases and ability to see a broader range of DNA sizes and GC-contents [24] and a wider range of expression levels [25] relative to amplification-based sequencing methods.

## Results

### cDNA Synthesis

For RNA Seq, cDNA was generated by priming RNA with random hexamers and extending with reverse transcriptase (RT) except for one experiment in which selected hexamers (avoiding sequences that prime rRNA) were used [26]. For DGE experiments, total RNA was used as starting material and an oligo dT-based primer was used for cDNA priming [11], [27]. First strand cDNA was tailed with terminal transferase and dATP. This tailed cDNA was sequenced after hybridization to dT50 covalently bound to a flow cell surface. No amplification, ligation or size selection was necessary, minimizing opportunities for introducing methodological biases. Each cDNA sample was sequenced on one or more channels of a HeliScope Genetic Analysis System with the resulting reads filtered for length (≥25 nt) and base addition order artifacts. The remaining reads were aligned to known transcripts as defined by UCSC Genes track as well as the complete human genome for unannotated transcripts.

### Read Counting and Normalization

Because ribosomal RNAs (rRNA) and mitochondrial RNAs (mitoRNA) are so common but generally of less interest than other transcripts, they were removed prior to analysis. To compare expression levels across samples that have differing numbers of reads, it is necessary to normalize the total number of reads to a constant value. This is accomplished by multiplying the filtered read counts from all non-rRNA/mitoRNA reads by a constant factor to generate a total of 1,000,000 reads per sample. Expression is thus given as Reads Per Million (RPM). Elsewhere, this is frequently further adjusted to Reads per thousand (K) nucleotides Per Million reads (RKPM). As discussed later, this additional normalization introduces a variety of problems so, except for spike-in data, all comparisons will be made using RPM rather than RPKM.

### RNA Fractionation and its impact

With total RNA, >80% of aligned reads correspond to rRNA, reducing the number of reads arising from transcripts of higher interest. Because the precision with which any RNA Seq measurement can be made is directly dependent on the number of times each transcript is counted, eliminating rRNA from sequencing allows a much higher number of counts to be obtained from all other RNAs. However, selective removal of any class of RNA introduces the potential risk of inadvertently altering the concentration of other RNAs [28] so an understanding of the impact of selection procedures is required. The most common selections carried out for RNA Seq experiments include depletion of rRNAs, often using a RiboMinus kit (Invitrogen), or selection of polyA RNA using oligo-dT binding. Additionally, selective priming can be performed either using primers specific for certain classes of RNA or using an oligo-dT primer as with DGE measurements. PolyA RNA selection can be accomplished in multiple ways, generally using oligo-dT attached to magnetic beads, plates, or cellulose.

To assess the impact of RNA selection on RNA Seq results, total RNA from human liver, human brain, and K562 cells, derived from human leukemia cells [29], was either sequenced directly, sequenced after RiboMinus rRNA depletion, or sequenced after one or more rounds of polyA selection. Ribosomal/mitochondrial reads ranged from 80–88%/2–8% for total RNA, 47–77%/4–15% for RiboMinus RNA, and 9–25%/10–25% for singly-selected polyA RNA from the three sources of RNA (Table 1). The contribution of mitochondrial reads rises with increased polyA selection because those transcripts are also polyadenylated [30] and thus enriched as more rRNA is removed.

**Table 1:** Summary of sequence data for analyzed channels.

Tissue/Cell	RNA Fractionation	Other	# Chan	Non ribo/mito Reads	Mito Reads	Ribo Reads	Total Reads	% Non ribo/mito	% Mito	% Ribo
K562	polyA		4	30,456,413	6,600,803	5,518,076	42,575,291	71.5	15.5	13.0
K562	RiboMinus		4	9,710,364	2,757,107	20,091,962	32,559,433	29.8	8.5	61.7
K562	Total		3	1,742,177	925,339	22,116,669	24,784,185	7.0	3.7	89.2
K562	DGE		1	2,785,898	671,865	2,908,141	6,365,904	43.8	10.6	45.7
Liver	polyA		12	59,005,359	26,439,549	34,136,252	119,581,159	49.3	22.1	28.5
Liver	RiboMinus		6	5,785,654	2,988,093	43,449,859	52,223,606	11.1	5.7	83.2
Liver	Total		9	4,673,697	5,964,424	81,889,062	92,527,182	5.1	6.4	88.5
Liver	DGE		2	9,124,996	3,377,497	4,770,979	17,273,472	52.8	19.6	27.6
Liver	polyA	Frag	4	36,957,011	14,711,900	19,748,540	71,417,451	51.7	20.6	27.7
Liver	DGE	Frag	2	12,074,196	5,126,958	6,533,234	23,734,388	50.9	21.6	27.5
Liver	polyA cellulose 1×		2	8,119,632	5,290,388	7,932,243	21,342,264	38.0	24.8	37.2
Liver	polyA cellulose 2×		2	7,040,565	3,833,472	454,005	11,328,042	62.2	33.8	4.0
Liver	Flow Through		2	412,972	531,004	14,474,302	15,418,277	2.7	3.4	93.9
HL60	polyA		1	4,001,738	466,516	782,963	5,251,217	76.2	8.9	14.9
HL60	polyA	tRet	1	8,678,984	2,031,609	1,806,003	12,516,596	69.3	16.2	14.4
HL60	Total		3	1,136,662	792,147	14,393,135	16,321,944	7.0	4.9	88.2
HL60	Total	tRet	3	935,863	1,441,219	12,884,604	15,261,686	6.1	9.4	84.4
HL60	DGE		1	4,448,499	509,554	1,183,895	6,141,947	72.4	8.3	19.3
HL60	DGE	tRet	1	3,919,856	1,808,924	3,955,218	9,683,999	40.5	18.7	40.8
HL60	Selected Primers		3	5,266,758	1,357,640	7,965,596	14,589,995	36.1	9.3	54.6
Brain	polyA		4	6,883,542	5,788,016	4,847,539	17,519,097	39.3	33.0	27.7
Brain	RiboMinus		4	2,993,303	4,328,560	26,472,617	33,794,480	8.9	12.8	78.3
Brain	Total		2	918,388	1,686,869	17,460,401	20,065,658	4.6	8.4	87.0
Brain	DGE		1	540,681	1,194,013	909,609	2,644,303	20.4	45.2	34.4

### Analysis of K562 RNA

For K562 cells, three channels of total RNA, two channels each for two replicate samples of RiboMinus RNA prepared in parallel, and duplicate single channels of two replicate samples of polyA RNA were compared. The UCSC reference transcriptome included 28,808 non-ribosomal, non-mitochondrial annotated transcripts, each representing a collapsed set of transcript annotations. Transcript counts for all channels used in this analysis can be found in the GEO database under accession GSE28123 and sequence reads are available at the Sequence Read Archive under accession number SRP006040. The 69 individual samples available are listed in Table S1. To avoid the common practice of artificially inflating correlation coefficients with non-expressing genes, annotated transcripts were included in this analysis only if present at >5 RPM in any of the compared samples. For K562 samples, 16,830 such transcripts were included based on this cutoff. Whenever different channels of the same sample were combined for analysis, the read counts were summed prior to normalization. Replicates of the same RNA sample run on different flow cell channels or different HeliScope sequencers were found to be nearly indistinguishable (R>0.99, all correlations presented are Spearman, see Text S1 for more detailed discussion).

**Figure 1:**
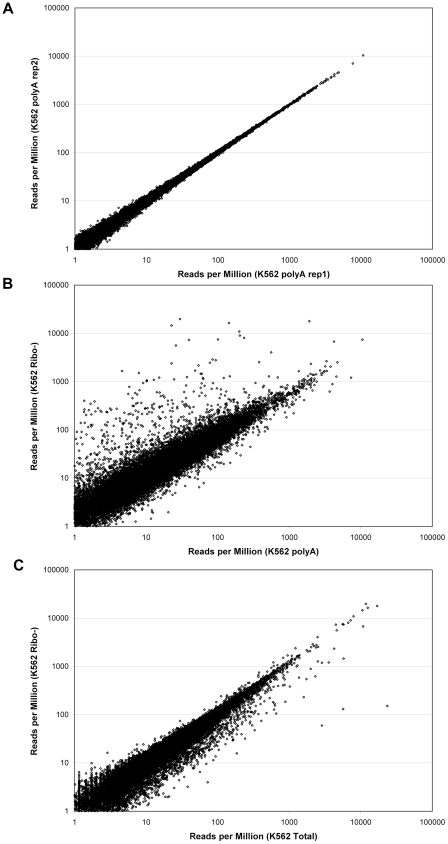
Comparison of K562 RNA selected in different ways. The same sample of total K562 RNA was used to prepare polyA RNA and RiboMinus RNA as described in Materials and Methods. The normalized RPM was then compared on a log-log plot for two replicate samples of polyA RNA (A), polyA RNA relative to RiboMinus RNA (B), and RiboMinus RNA relative to the starting Total RNA (C).

When different samples of K562 polyA RNA were compared to each other, it did not matter whether the duplicate runs or replicate samples were compared as the correlations were >0.99 in all pair-wise comparisons (Table 2). One pair of polyA replicates is shown graphically in Figure 1A. There are no transcripts that are significant outliers in this comparison. Though there is broadening below ∼10 RPM, this is expected from the stochastic nature of counting low frequency transcripts. In marked contrast to the high correlation when polyA RNA is compared to itself, there is a much broader distribution and many outliers when polyA RNA is compared to RiboMinus RNA (R = 0.85–0.88, Figure 1B). Most outliers have significantly higher RPM in the RiboMinus sample, as would be expected if the polyA method is selecting out a subset of RNAs and eliminating more than simply rRNA. The RiboMinus depletion method eliminates fewer RNAs than polyA selection, leaving a larger number of transcripts in the overall pool. Many of the most significant outliers are non-coding RNAs which are not expected to be polyadenylated and would not be removed using methods designed to eliminate only rRNA. Comparison of total K562 RNA with the K562 RiboMinus RNA shows an intermediate level of correlation (R = 0.94–0.95) with far fewer outliers (Figure 1C). The high number of ribosomal reads in the total RNA sample lowers the precision of those measurements. It is also worthy of note that RiboMinus depletion of RNA samples is dependent on the quality of the starting RNA. Because a relatively small number of probes (8 total probes for all rRNAs) is used for depletion of the large rRNAs, even mildly degraded RNA samples are not depleted well because many partial fragments are not targeted. With such samples, we have used 30 evenly-distributed probes and this is much more effective at removing rRNA (data not shown). Similarly, the commercial RiboZero kit (Epicentre) is much more effective at removing rRNA, typically leaving less than 10% rRNA reads (data not shown).

**Table 2:** Correlations among K562 RNA samples.

K562	Total	RiboMinus rep1	RiboMinus rep2	polyA rep1A	polyA rep2A	polyA rep1B	polyA rep2B
Total		0.95	0.94	0.82	0.81	0.82	0.82
RiboMinus rep1			0.98	0.86	0.85	0.86	0.86
RiboMinus rep2				0.88	0.88	0.88	0.88
polyA rep1A					0.99	1.00	1.00
polyA rep2A						0.99	1.00
polyA rep1B							1.00

### Analysis of Liver RNA

**Figure 2:**
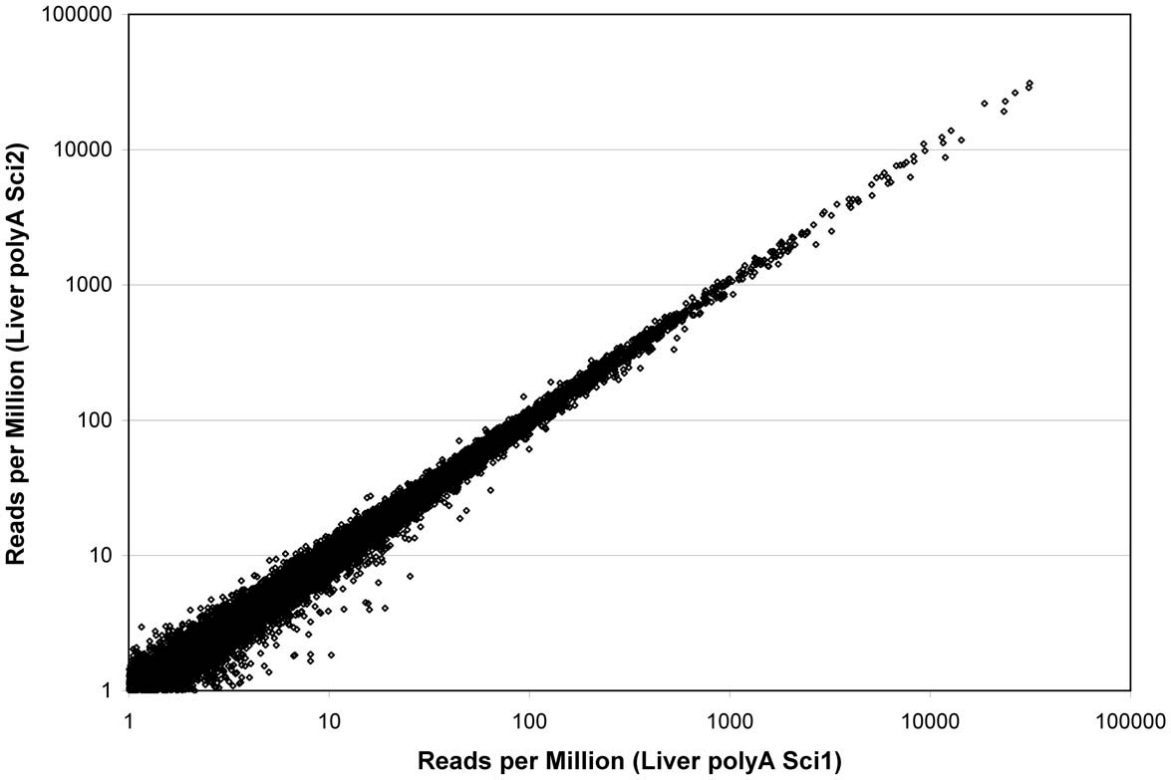
Comparison of liver polyA prepared by two individuals. The same sample of total liver RNA was used to prepare polyA by two different individuals as described in Materials and Methods. The normalized RPM for all transcripts with greater than 1 RPM is shown on a log-log plot.

To determine the generality of the patterns observed with the K562 samples, the same analysis was repeated with liver RNA (Table 3). With the 5 RPM cutoff, 17,204 transcripts were included. The correlations between all pairs of liver RNAs are somewhat lower than the corresponding K562 pairs which is likely caused by the difference in expression patterns between the two samples. The liver sample has more transcripts with very high or very low expression levels (Table 4). Low expressing transcripts are inherently more variable. However, the same pattern of polyA samples being highly correlated with each other (0.98–0.99) and much less correlated with RiboMinus (0.60–0.74) and total RNA (0.63–0.71) samples is maintained. To further test technical replication, polyA RNA was selected and processed independently by two scientists. polyA rep1 and rep2 were made by one scientist and polyA rep3, rep4, and rep5 were made by a different scientist. The pair-wise correlations were 0.99 for samples prepared either by the same or by different scientists (Figure 2). Thus, highly reproducible results can be obtained when the same protocol is carried out independently.

**Table 3:** Correlations among Liver RNA samples.

Liver	RiboMinus Scientist 1	RiboMinus Scientist 2	polyA Scientist 1	polyA Scientist 2	polyA Fragmented	DGE	polyA1 cellulose	polyA2 cellulose
Total	0.93	0.92	0.70	0.67	0.67	0.71	0.71	0.63
RiboMinus Scientist 1		0.93	0.73	0.62	0.70	0.75	0.74	0.66
RiboMinus Scientist 2			0.67	0.63	0.63	0.69	0.68	0.60
polyA Scientist 1				0.99	0.99	0.87	0.98	0.97
polyA Scientist 2					0.99	0.86	0.98	0.98
polyA Fragmented						0.86	0.98	0.98
DGE							0.86	0.83
polyA1 cellulose								0.97

**Table 4:** Number of expressed collapsed transcripts.

	5–10	>10–50	>50–100	>100-1K	>1K–10K	>10K	Total
Total Liver	4378	8155	1477	1114	89	11	15224
Ribo- Liver	4399	8078	1392	1082	91	10	15052
polyA Liver	3992	6365	1076	1038	90	11	12572
DGE Liver	4332	6872	996	1007	95	10	13312
polyA 1× Liver	4069	6294	1048	1004	89	12	12516
polyA 2× Liver	3740	5663	1004	1024	90	13	11534
Total K562	3199	8697	2153	1710	57	6	15822
Ribo- K562	3101	8360	2171	1716	65	5	15418
polyA K562	2878	6925	2231	1901	105	1	14041
DGE K562	3087	7476	1848	1497	131	2	14041
Total Brain	3803	10196	2503	1667	48	3	18220
Ribo- Brain	3848	10357	2506	1598	49	3	18361
polyA Brain	3733	9092	2204	1819	54	5	16907
DGE Brain	3290	10031	2394	1692	55	4	17466

The polyA samples discussed thus far were prepared using a single selection with oligo-dT magnetic beads. Another common method involves one or two selections using oligo-dT cellulose. According to the product manufacturers, the oligo-dT on the beads we used was 25 nt and the oligo-dT on the cellulose was a mix ranging from 12–25 nt. polyA RNA selected once with oligo-dT cellulose shows a similar amount of rRNA (33%) to polyA RNA selected once with beads. A second round of oligo-dT cellulose selection yielded a sample with only 3.6% rRNA. As shown in Table 3, the correlation of total or RiboMinus RNA with polyA RNA selected once with cellulose (R = 0.71–0.74) is about the same as polyA RNA selected once with beads (R = 0.62–0.73). The twice-selected RNA correlates less well (R = 0.63–0.66), presumably due to a more efficient removal of non-polyadenylated RNAs that are still present in the total and RiboMinus samples. If one examines the RNA that flows through the column during the second round of polyA selection, it has a higher proportion of rRNA (89%) than the starting sample but also contained significant residual amounts of polyA RNA. Most of the differences between the once and twice selected RNAs (Figure S1) occur in transcripts that also change markedly on going from total RNA to polyA RNA.

The non-rRNA reads in the flow through mirror what is selected but there are also interesting differences. Most of the reads that are underrepresented in the second selection relative to the flow through are either short non-coding RNAs or very long mRNAs. The former may have shorter than average polyA tails and hence less likely to stably bind the matrix. Many are detected in DGE experiments, suggesting some level of polyadenylation. The very long RNAs are probably overrepresented in the flow through because they are more likely to suffer degradation during processing. Conversely, the transcripts that tend to be underrepresented in the flow through include short, ribosomal protein encoding mRNAs that are probably less susceptible to degradation. Transcripts that show the greatest variation among the different polyA selection strategies are those that change most dramatically upon examination of total RNA versus any polyA RNA.

### Selected Primers and spikes

Another indirect method for elimination of rRNA reads has been described [26] in which cDNA is primed using non-random hexamers, selected to avoid reverse transcription of rRNA. This approach was shown to achieve markedly lower numbers of rRNA reads with non-rRNA going from 22% with random hexamers to 87% with non-random hexamers with brain RNA [26]. We carried out the same approach with HL60 total RNA to determine the performance of cDNA made with selected primers relative to cDNA made after rRNA depletion or polyA selection. The use of selected primers caused the desired reduction in rRNA reads non-ribosomal reads improving from 11% with random hexamers to 45% with selected hexamers. However, the impact on the non-rRNA reads was not uniform with differential effects on many transcripts. It is possible that even better specificity versus rRNA could be obtained with optimized hybridization conditions. As a class, the most affected RNAs were the very short, non-coding ones (less than 200 nt) though, there were widespread effects on other transcripts as well (Figure S2, R = 0.75).

Certain samples were also spiked with a set of seven RNAs produced by *in vitro* transcription and combined in known absolute proportions [6]. These RNAs were added to a liver polyA sample prior to cDNA synthesis and analyzed as part of the overall experiment. When the known lengths of these RNAs were accounted for, their detected abundance was well correlated with the proportions in the initial sample (R>0.99, see Text S1 and Figure S3 for additional details).

### Fragmentation

Fragmentation of RNA and/or cDNA has been used previously [6] to achieve more even sequence coverage throughout the length of transcripts. This has the benefit of allowing more exons to be detected with the same number of reads and potentially lessening the impact of secondary/tertiary structures on priming efficiency in very long RNAs. However, the effect that fragmentation may have on transcript representation has not been previously determined. As shown in Figure S4, there is a very high correlation (R = 0.99) between fragmented/unfragmented sample pairs though there are more outliers at high and medium expression levels than expected for truly identical samples. There is a trend for the fragmented protocol to have lower expression for shorter RNAs. Many of these are non-coding RNAs and shorter coding transcripts such as ribosomal proteins. These classes of transcripts will be lost or underrepresented using protocols that include size selection or fragmentation. As shown in Figure S5, there is very little impact on transcripts longer than 1 kb but the impact becomes increasingly significant below that length. Overall, for all transcripts with >100 RPM in either sample, the ratio of unfragmented to fragmented expression is weakly correlated with log(median length) (R = −0.38). Transcripts of less than 1000 nt have a median 1.3 fold higher expression level when comparing unfragmented to fragmented transcripts.

### Digital Gene Expression

In addition to RNA Seq methods in which reads are captured from throughout the transcript length, it is also possible to use tag-based approaches in which reads are obtained only from the 5′ or 3′ end of each transcript. This should lead to improved counting though at the expense of characterizing splice and other sequence variants. Single-molecule Digital Gene Expression (DGE) has been described using yeast polyA RNA [11]. Total RNA is used which minimizes sample handling. A primer is hybridized to the polyA tail and then reverse transcribed to the end of the RNA. For relatively short RNAs (<2 kb), most molecules are extended to the 5′ end. Many longer RNAs are also fully extended but random cleavages that occur during sample processing and less than perfect RT processivity lead to stops throughout the RNA. As with RNA Seq, first strand cDNA is polyA tailed. With DGE, the large fraction of reads arising from the 5′end make it easier to count as well as provide a direct experimental determination of the initiation site.

**Figure 3:**
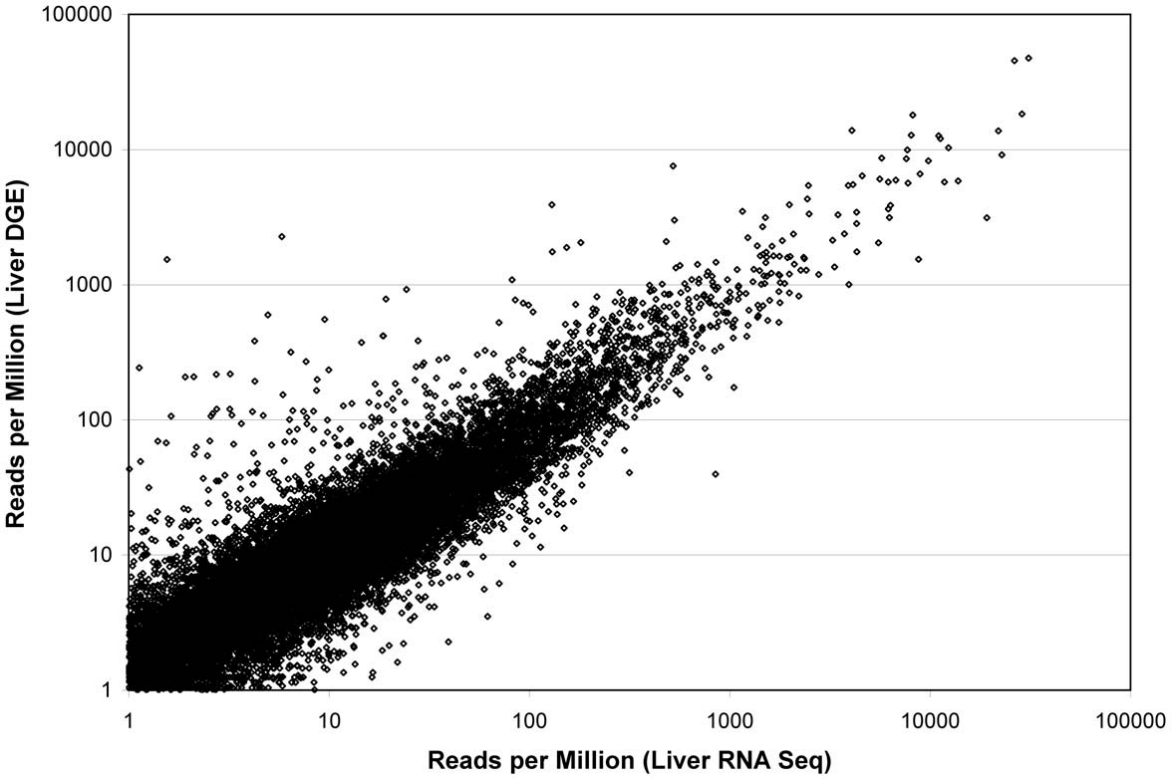
Comparison of liver DGE vs. RNA Seq polyA. The same sample of total liver RNA was used directly for DGE or was polyA purified. The resulting cDNA for the two methods was then tailed and sequenced as described in Materials and Methods. The normalized RPM for all transcripts with greater than 1 RPM is shown on a log-log plot.

Because DGE should produce one read rather than many reads per RNA molecule, the agreement between the two methods is not perfect (Figure 3). DGE also detects a greater number of genes relative to polyA samples and this is true for all tissues/cells tested. A large part of this is due to non-coding RNAs and short RNAs that are not well detected by RNA Seq, most likely due to the small number of priming sites in short RNA. With DGE, all polyadenylated transcripts, short or long, will have a single priming site and thus detected in their proper proportions. If all the genes that are expressed at 50 RPM or higher in any of the liver RNA Seq or liver DGE samples are examined for the most extreme differences in expression ratios, 14 genes are found to be >100× higher in DGE than RNA Seq while none is that extreme in the other direction. Of the 14 genes present at high amounts in DGE, nine are short, non-coding RNAs. The most extreme over-expressers in DGE are the snoRNAs NR_002955, NR_002956, and NR_002973 which are found at >1000 RPM in DGE while present at less than 6 RPM in RNA Seq.

### Transcriptome Complexity

A noticeable impact of the double polyA selection is a loss in complexity of the sample with many genes missing. It is pronounced even at the level of annotated transcripts and has been shown to be even more extreme among unannotated transcripts [28]. The bead-selected liver polyA sample has about 2500 fewer transcripts than either the total or RiboMinus sample and is very similar to the 1× cellulose selected sample in terms of transcript count (Table 4). The 2× oligo-dT cellulose-selected sample lacks an additional 1038 transcripts beyond the reduced number obtained with the bead-selected RNA.

### Differential Expression

While examination of static gene expression levels is instructive, it is generally of more interest to study changes in gene expression. However, care must be taken in such analyses. For example, it has been previously noted [31] that differential expression differences are length dependent when determined via RNA Seq though not with microarrays. This can be partially explained by the increased sampling of longer transcripts which reduces the variation and allows smaller expression differences to be observed. To compare our results with the previous study, a similar analysis was performed. Liver and K562 polyA RNA were compared for significant expression differences using a 2-sided T-test with a multiple testing correction factor for the 18335 genes with at least 5 RPM in any sample. Because of the different tissue sources, a large number (5239) of transcripts was found to be differentially expressed. The same analysis of liver polyA prepared by different scientists yielded only 5 of 13910 transcripts as being differentially expressed after correction.

**Figure 4:**
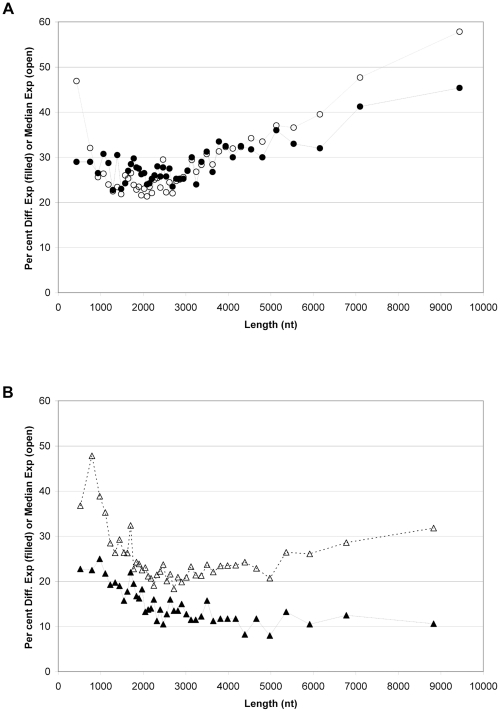
Length dependence of differential expression. Transcripts were divided into bins of 400 in order of increasing length for all transcripts. Four separate channels each of liver and K562 polyA for RNA Seq (A) and two separate channels for DGE (B) were averaged and all transcripts with greater than 5 RPM in either liver or K562 total RNA were analyzed for differential expression. In each set, two-sided t-tests were performed and the resulting differences examined for statistical significance. With both RNA Seq and DGE, the individual significance results were corrected for multiple testing by dividing the raw significance by the number of transcripts in the analysis. The number of transcripts in each bin that was differentially expressed was then used to calculate per cent differential expression. This value is lower for DGE than for RNA Seq due to the lower number of channels analyzed. Additionally, the median expression level for all transcripts in each bin was calculated and plotted in RPM.

Transcripts were binned by length in groups of 400 and the fraction of genes that were differentially expressed plotted versus mean transcript length of the bin. As observed previously with other sequencing platforms and expected based on increased sampling, a higher proportion of long transcripts are observed to be differentially expressed (Figure 4A). However, amplification-based sequencing platforms suffer from a sharp drop in detection of differential expression below 1000 nt, likely due to their difficulties in detecting short transcripts [31], that is not observed with this data set. If expression levels were constant as a function of length, one would expect a continued drop in detection of differential expression below 1000 nt because of the shorter length over which sampling is possible. However, the shorter RNAs include many highly expressed transcripts that skew the median expression level of the shorter bins. The 233 differentially expressed transcripts in the two shortest bins include 45 non-coding RNAs, 35 histones, and 9 ribosomal proteins. In contrast, DGE analysis of the same samples yields a much more even length dependence above 1000 nt (Figure 4B) because only a single read is generated from each transcript. The increased expression levels of transcripts less than 1000 nt leads to higher levels of detectable differential expression with DGE.

Comparison of a tissue with a cell line provides a rich source of differentially expressed transcripts but may not adequately reflect the issues faced with systems of greater biological interest. For a more relevant model, RNA was prepared from HL60 cells growing in standard growth media or growing in the presence of t-retinoic acid (t-Ret) to induce differentiation [32]. Many transcripts are dramatically changed and there is a high level of consistency among the different RNA samples when examining those that are markedly altered (see Text S1). Among the most significant gene expression changes are the downregulation of *c-Myc* and the upregulation of *THBS1* and *CTSD*, all of which change by more than 1000 RPM and greater than 20×.

The importance of looking beyond just polyA RNA is highlighted by our observations of differential expression with many non-coding and histone RNAs in total RNA. Histone mRNAs are known to lack a polyA tail and their 3′ ends are processed by a variety of proteins including Stem Loop Binding Protein [33]. Of the 43 histone transcripts with an expression level >100 RPM in total RNA from HL60 cells, all are downregulated by t-Ret treatment, by an average 2.2×. As expected due to the lack of polyA tail, only one gene shows an expression level >100 RPM in the polyA and DGE samples and there are no consistent expression changes among the entire set of histones in those samples. *SLBP* is also downregulated 2.9× with t-Ret treatment in total RNA and 1.9× each in polyA or DGE samples. Thus, these expression changes, which are similar to other conditions in which DNA replication in cells ceases [34], are entirely missed when only the polyA fraction is examined. Similarly, the non-coding RNAs NR_002955, NR_002970, and NR_002977 are all found at less than 2 RPM in both polyA samples but are present with >20 RPM in both untreated RiboMinus samples and are increased at least an additional 5× by t-Ret treatment.

### Genomic Bin analysis

**Figure 5:**
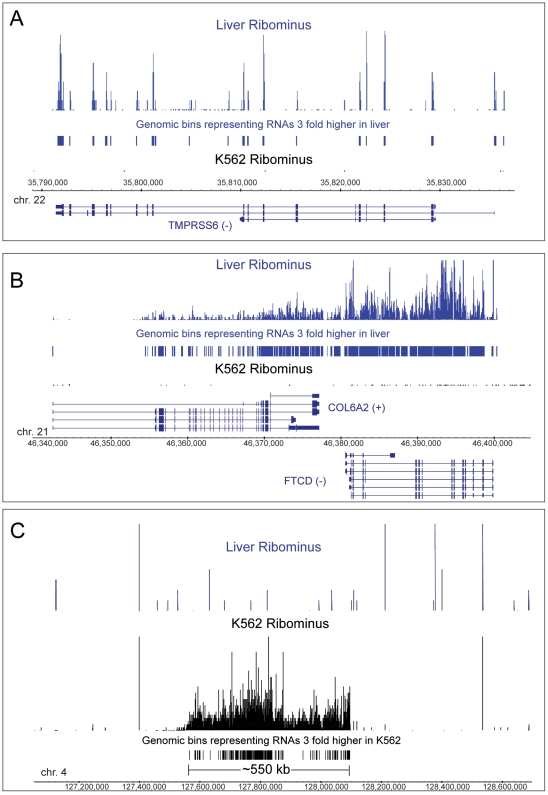
Analysis of expression using genomic bins. Three different genomic regions (A, B, C) are compared for expression with RiboMinus depleted RNA from liver and K562 samples. The vertical bars of varying heights show the expression level in each bin and the constant height bars designate those bins which are over-expressed at least 3× in either sample. Known annotated genes are labeled. In C, there is a 550 kb region with no annotated genes which is highly expressed in K562 but not liver and corresponds to a very long intergenic region (vlinc) on chromosome 4 [28]. The corresponding chromosomes and the coordinates of the regions (hg18 version of the genome) are shown.

Because of the limitations imposed by incomplete annotation of the transcriptome and oversampling of longer transcripts, we have explored other methods for analyzing expression. By examining bins of uniform size across the whole genome, it is possible to eliminate both the effect of transcript length and the issue of collapsing a complex set of overlapping and poorly characterized transcripts into a master list. As an example, we compared K562 and liver RNA samples (Figure 5). The genomic sequence was split into 100 bp bins and the number of reads in each bin counted. The K562/liver ratio of reads was calculated for each bin that had at least 0.95 RPM (corresponding to at least 3 reads prior to the normalization in the sample with the smallest number of reads, liver). 69,890 exonic, 12,929 intronic and 9,438 intergenic bins were identified corresponding to transcripts expressed >3× higher in K562. For liver RNA, 36,679 exonic, 26,570 intronic and 18,464 intergenic bins were >3× more highly expressed. The increased number of more highly expressed intronic and intergenic bins in liver occurred despite the fact that the liver sample had a lower fraction of non-exonic reads than K562 and suggests that non-exonic reads tend to be more clustered in liver and more dispersed in K562.

## Discussion

Measurement of RNA expression levels has been critical for understanding many pathways and biological systems. Up until recently, it has only been possible to examine a small number of transcripts over a wide range of expression levels using qPCR or a large number of transcripts over a narrower range of expression levels using microarrays. With the advent of RNA Seq and DGE technology, it is now possible to measure both a wider dynamic range of expression and in an unbiased manner at a genome-wide scale, a combination unachievable with any other technology. With the exquisite sensitivity and precision now made possible by next generation sequencing technologies, choice of sequencing platform, sample preparation methods, and the nature of the source RNA are increasingly important in setting the overall precision and reproducibility of the measurements allowed by this technique. Thus, these choices can significantly influence the results obtained.

Measurement of expression levels for transcripts having a high number of reads is inherently more precise than measuring transcripts with fewer reads. However, when using random hexamer priming for generating cDNA, the number of reads arising from a given transcript depends not only on the number of such transcripts in a cell but also on its length because longer RNAs have more opportunities for priming (and hence more reads). Thus, the absolute number of reads is frequently further adjusted to Reads per thousand (K) nucleotides Per Million reads (RKPM). Unfortunately, this adjustment eliminates the simple relation between read count and precision. Furthermore, any length normalization is imperfect because so many annotated loci have multiple transcripts of different lengths. Length normalization is intended to fix the differences in the number of hexamer priming sites, but this is not an ideal surrogate as RNA secondary structure and GC content also play major roles in priming efficiency [6].

Furthermore, attempting to correct RNA Seq data for length increases the correlations among all pairs of datasets, not just those that should be dependent on length. As discussed in Text S1, normalization of data using randomized transcript lengths rather than real lengths also results in an increased correlation relative to unnormalized data (Figure S6), demonstrating the presence of a mathematical artifact in this approach. While there are ways to reduce this artificial induction of correlation caused by length “correction”, they do not take into account whether there may actually be a biological basis for a correlation between length and expression. To avoid the issue of inducing false correlations, we have used RPM rather than RPKM except for spike analysis.

One method for avoiding overcounting of long transcripts is the use of a tagging approach in which only a single read arises from each transcript. This eliminates many of the counting issues introduced by multiple tag approaches but reduces the evenness of coverage. The DGE protocol generates a single read predominantly from the 5′ end of the transcript via selective priming from the 3′ end [11], providing a direct indication of transcription initiation. Even though DGE reads arise from polyadenylated RNA within the total RNA starting material, DGE detects more genes than found with standard polyA RNA preparations (Table 4). Thus, the choice of DGE versus RNA Seq will depend on whether a researcher is more interested in overall gene expression or even coverage. Other tagging approaches such as SAGE and CAGE would have similar benefits for counting and minimizing rRNA reads without employing a separate fractionation step [13], [15].

RNA and cDNA fragmentation are frequently used to improve evenness of coverage across the length of transcripts so splice junctions and SNPs can be more efficiently detected. However, this improved coverage comes at the expense of reduced coverage of short RNA species. Short RNA species are produced from many locations [35], so the importance of more even coverage of exons must be weighed relative to the the cost of missing other RNAs. Comparing fragmented and unfragmented samples can be done but it must be with the realization that expression levels for short transcripts will be affected. Similarly, efforts to improve the precision of mRNA measurements by selecting the polyA fraction come at the expense of losing 25% of the annotated transcripts (Table 4) and far more unannotated transcripts [28]. Some transcripts can also yield highly variable results because they are differentially removed by polyA selection.

Total RNA and rRNA-depleted RNA have significantly more reads mapping to unannotated regions of the genome than does polyA RNA, suggesting they should be used as the source of RNA in transcriptome profiling if a comprehensive survey is desired. While rRNA-depleted samples still contain a significant number of rRNA reads, the fraction corresponding to non-rRNA, non-mitochondrial reads increases by several fold and allows sufficient precision to make differential expression experiments practical for most transcripts. Furthermore, we have observed that the use of a larger number of probes (30 versus 8) or other commercial kits such as RiboZero for rRNA removal significantly improves depletion (data not shown) thus enhancing the number of informative reads.

Annotated sequences provide genomic bounds to which one can either map the sequence reads directly or within which one can sum all the mapped reads to generate transcript counts. Unannotated transcripts do not offer this opportunity. Computational methods have been developed to reconstruct transcript structures based on overlapping reads [36]. However, such methods were based on data derived from polyA+ RNA, which has a much simpler expression profile in annotated regions compared to that of total RNA and it remains to be seen whether such methods would be useful for deconvoluting individual transcripts found in regions such as those in Figures 5B and 5C. An alternative method would be to split a genome into a set of overlapping bins of variable sizes. Differentially-expressed transcripts are detected based on the number of reads that fall within each bin in each sample. Different bin-sizes would preferentially detect different transcripts. For example, longer transcripts would benefit from larger bins as opposed to exons that would be better detected with shorter, exon-size bins. Additional experimentation will be required to characterize the transcripts underlying each bin. For example, high-throughput implementation of Rapid Amplification of cDNA ends [37] and 5′ and 3; ends as determined by tagging approaches could be used to better understand each genomic region.

There are also technical limitations that force particular choices on experimenters. Most next-gen sequencing platforms require amplification just prior to sequencing and, in many cases, also amplify cDNA earlier in the protocol. These steps can introduce bias based on GC-content and length. One recent protocol eliminated cDNA amplification [20] but still required RNA ligation as well as amplification for sequencing. Because the 5′ end of many RNAs is capped or otherwise modified, it is frequently not amenable to ligation without additional enzymatic treatment and thus these exons can drop out of the sequencing pool (data not shown). Technical issues caused by a protocol or sequencing platform may cancel out when transcripts are compared in a differential expression study even when the absolute expression levels are incorrect. However, the degree to which technical artifacts are reproducible and thus offset each other may vary. For example, one pair of libraries prepared and sequenced identically with the standard Illumina protocol [20] was not well correlated (R = 0.48 when calculated including only genes with at least one sample with >5 RPM compared to the inflated R = 0.82 when including non-expressed genes), unacceptably variable by most standards while another pair of libraries prepared using a different method was highly correlated (R>0.99). Thus, it cannot be assumed that technical issues will always cancel out.

While it would be desirable to ignore the costs of any experimental plan and make technical choices solely based on optimizing the experimental outcome, the reality is that the cost of sequencing and analysis means that a cost-blind approach is rarely possible. Thus, each technical choice must be a trade-off between cost and quality of results. For example, for a full view of RNA expression, sequencing polyA RNA results in the loss of a tremendous number of transcripts, but, use of total RNA can yield 5–25-fold fewer non-ribosomal, non-mitochondrial reads [28] which substantially reduces the precision of all expression values and limits the ability to detect low-expressing RNAs. If cost were no object, one could simply sequence more but the size of most experiments makes this prohibitively expensive. Use of rRNA depletion is a reasonable compromise to achieve lower cost while retaining comprehensiveness of coverage. In our original experience, many samples generated using RiboMinus depletion contain up to ∼50% rRNA reads, the more recent rRNA depletion procedures are much more efficient (see above) and thus more cost effective. Most importantly, such methods allow provide a more comprehensive view of the transcriptome at a cost similar to polyA selection. Similarly, choosing fragmentation of RNA or cDNA for detecting splice junctions or SNPs is more efficient but this limits the ability to see short transcripts. This situation is more complicated in amplification-based protocols because amplification efficiency can be an issue for both short and long RNAs.

There is no perfect set of technical choices for transcriptome analysis but, armed with the knowledge of the impact that various choices have on the outcome, experimenters can choose the best set of parameters to match their scientific needs and resources and realize the potential limitations in their analysis. RNA Seq, DGE and related methods provide valuable tools for biologists that allow detailed characterization of both the level of expression of known transcripts with unparalleled precision and the identification of novel transcripts. However, these techniques are subject to artifacts and biases, no matter what the sequencing platform or method of RNA processing. Less sensitive or comprehensive technologies have clear deficits with respect to what can be seen and not seen. The large amount of data generated by sequencing-based technologies can lull the experimenter into thinking one has a complete and accurate picture of gene expression. This is clearly not the case as technical choices affect the results. These choices and their associated caveats should be made knowingly so that conclusions can be drawn in as biologically-relevant a manner as possible while understanding the limitations of those conclusions.

## Materials and Methods

### RNA

Total RNA was obtained from commercial sources from the following cell lines and tissues: K562 (Ambion), normal liver and normal brain (Clontech), and HL60 (ATCC; CCL-240). The HL60 RNA was extracted from cells grown under 2 different conditions (cell treatment and RNA extraction by MIR Preclinical Services): 1. untreated, 2. treated with retinoic acid (RA) to induce differentiation (0.1%DMSO+1 µM RA for 5 days).

Before further fractionation, total RNA was treated with DNase I as follows: 50 µg of total RNA was mixed with 10 µl of 10× DNase I buffer (Roche), 2 µl of RNaseOut (Invitrogen) and 8 µl of recombinant DNase I (10 U/µl, Roche) and incubated for 45 minutes at 37°C. The RNA was then purified using the RNeasy MinElute kit (Invitrogen).

The DNase I-treated total RNA was either unfractionated (total RNA) or fractionated using one of the following methods: 1. Depleted of ribosomal RNA (rRNA) using the RiboMinus kit (Invitrogen) 2. polyA fraction was selected using a magnetic bead-based purification kit (Dynabeads mRNA purification kit, Invitrogen) or, 3. polyA fraction was selected using the oligo-dT cellulose method (Micro Poly(A)Purist Kit, Ambion). Some RNA samples were fragmented by heating at 95 C for 10 min prior to cDNA synthesis.

### Preparation of RNA for sequencing

100–400 ng of DNase I -treated RNA, except where noted, was mixed with the following reagents from the SuperScript III kit (Invitrogen). First 10 µl of 50 ng/µl Random Hexamers and 2 µl of 10 mM dNTPs were added in the total volume of 25 µl. When employing selected primers, the same conditions were used. The mixture was then placed in a thermocycler and heat denatured at 65°C for 5 min followed by rapid cooling on ice. Next, 5 µl of 10× cDNA synthesis buffer, 5 µl of 0.1 M DTT and 10 µl of 25 mM MgCl_2_ were added. The samples were returned to the thermocycler and allowed to incubate at 15°C for 20 min. Then, 2.5 µl of RNaseOut and 2.5 µl of SuperScript III reverse transcriptase were added and the samples were incubated at 25°C for 10 min, 42°C for 40 min, 55°C for 50 min and 70°C for 10 min.

After reverse transcription, RNA was removed by adding 1 µl of RNaseH (Invitrogen) and 1 µl of RNase If (New England BioLabs) to each sample and incubating at 37°C for 30 min. The cDNA was then purified by two rounds of purification over Performa columns (EdgeBio) and quantified using a NanoDrop spectrophotometer.

Next, a 3′ poly-A tail was added to the cDNA samples. cDNA (100 ng) was mixed with a control oligo to monitor tail length and water in a total volume of 33.5 µl. The mixture was denatured at 95°C for 5 min followed by rapid cooling on ice. 5 µl of 2.5 mM CoCl_2_, 5 µl of 10× terminal transferase (TdT) buffer (New England BioLabs), 5 µl of 50 µM dATP and 1.5 µl of TdT (20 U/µl, New England BioLabs) was then added and the samples were incubated at 42°C for 1 hr, and at 70°C for 10 min.

The 3′ ends of the polyA-tailed cDNA were then blocked with biotin-ddATP. The sample was denatured at 95°C for 5 min followed by rapid cooling on ice. 0.3 µl of 1 mM biotin-ddATP (Perkin Elmer) and 1.5 µl of TdT were added followed by incubation at 37°C for 45 min and 70°C for 10 min.

The control oligo was removed by digestion with the USER enzyme (New England BioLabs). 1 µl of the USER enzyme (1 U) was added to the sample and incubate at 37°C for 30 min.

The sample was then purified using AMPure beads (Agencourt) by bringing the volume up to 60 µl with water and adding 72 µl of the AMPure beads followed by incubation at room temperature for 30 min with agitation. The beads were then captured on a magnetic stand and washed twice with 70% ethanol. The beads were then allowed to air dry for 5–7 min, resuspended in 20 µl of water and left open for 30 min on the magnet. The eluate was collected, the beads were resuspended again in 20 µl of water and left for 5 min on the magnet. The eluate was collected again and combined with the first eluate.

Typically, the samples were hybridized to the HeliScope flow cell at a loading concentration of 100–350 pM.

Digital Gene Expression was performed as described [11].

## Supporting Information

Figure S1 **Varying methods of polyA selection.** polyA selection of liver RNA was carried out as described in Materials and Methods using either beads or cellulose. Expression levels for once-selected RNA are compared for beads and cellulose (A). Additionally, a fraction of the polyA RNA selected once with cellulose was selected again with cellulose to generate highly selected polyA RNA. The expression differences between once and twice selected RNA are shown (B).
(TIF)

Figure S2 **Artifactual correlations induced by length corrections.** Four different liver RNA samples were compared pair-wise for correlations with varying methods of correcting for transcript length.
(TIF)

Figure S3 **Selected versus random hexamer priming in HL60 total RNA.** cDNA was synthesized from HL60 Total RNA using either random hexamers or hexamers selected to avoid cDNA synthesis from ribosomal RNA. Expression levels for transcripts with more than 5 RPM in either sample are shown on a log-log plot.
(TIF)

Figure S4 **Fragmented versus unfragmented liver polyA.** polyA RNA was prepared from liver. Prior to cDNA synthesis, RNA was fragmented by heating to 95° in MgCl_2_. RNA was then prepared identically and sequenced. RPM for fragmented and unfragmented RNA is shown on a log-log plot.
(TIF)

Figure S5 **Ratio of expression for unfragmented/fragmented liver polyA RNA.** Liver RNA was fragmented after polyA selection as described in Figure S1. The ratio of expression was determined for all transcripts with greater than 50 RPM in either sample. Transcripts were then binned based on the UCSC median transcript length and average and median ratios of expression determined for each bin.
(TIF)

Figure S6 **Spike-in expression.** Spike-in RNAs were provided by Dr. Brian Willi in Dr. Barbara Wold's laboratory. These were added to a liver RNA sample and prepared as described above. Because the absolute number of molecules in the sample was known, the counts were adjusted for the known lengths and then plotted versus the known spike-in concentration. The number of transcripts per nanoliter of spike is shown on the horizontal axis and RPKM on the vertical axis. The single point below the diagonal is AGP which is only 325 nt long and represented by 3 reads and thus the least precise measurement among the spiked RNAs, reinforcing the issues with converting RPM to RPKM. Although two transcripts have lower RPKM than AGP, they are, in fact represented by more reads (19 and 7, respectively) because they are much longer (9786 and 1451 nt).
(TIF)

Table S1 Sequencing data summary. The sequence data channels used in the analyses and various figures and tables are listed with the machine used for sequencing, run date, and channel number. The figures in which each channel are included on the X or Y axis are noted as such or, if included in some other fashion, by “Yes”.
(XLS)

Text S1 Additional details on how sequence reads were aligned and counted, how normalization and correlations of data were carried out, and how spikes and expression differences were analyzed is provided.
(DOC)
