# Supplementary figures and images for: Protocol Dependence of Sequencing-Based Gene Expression Measurements
Source: PLoS One. 2011 May 6;6(5):e19287. doi: 10.1371/journal.pone.0019287 (PMC3089619; doi:10.1371/journal.pone.0019287)

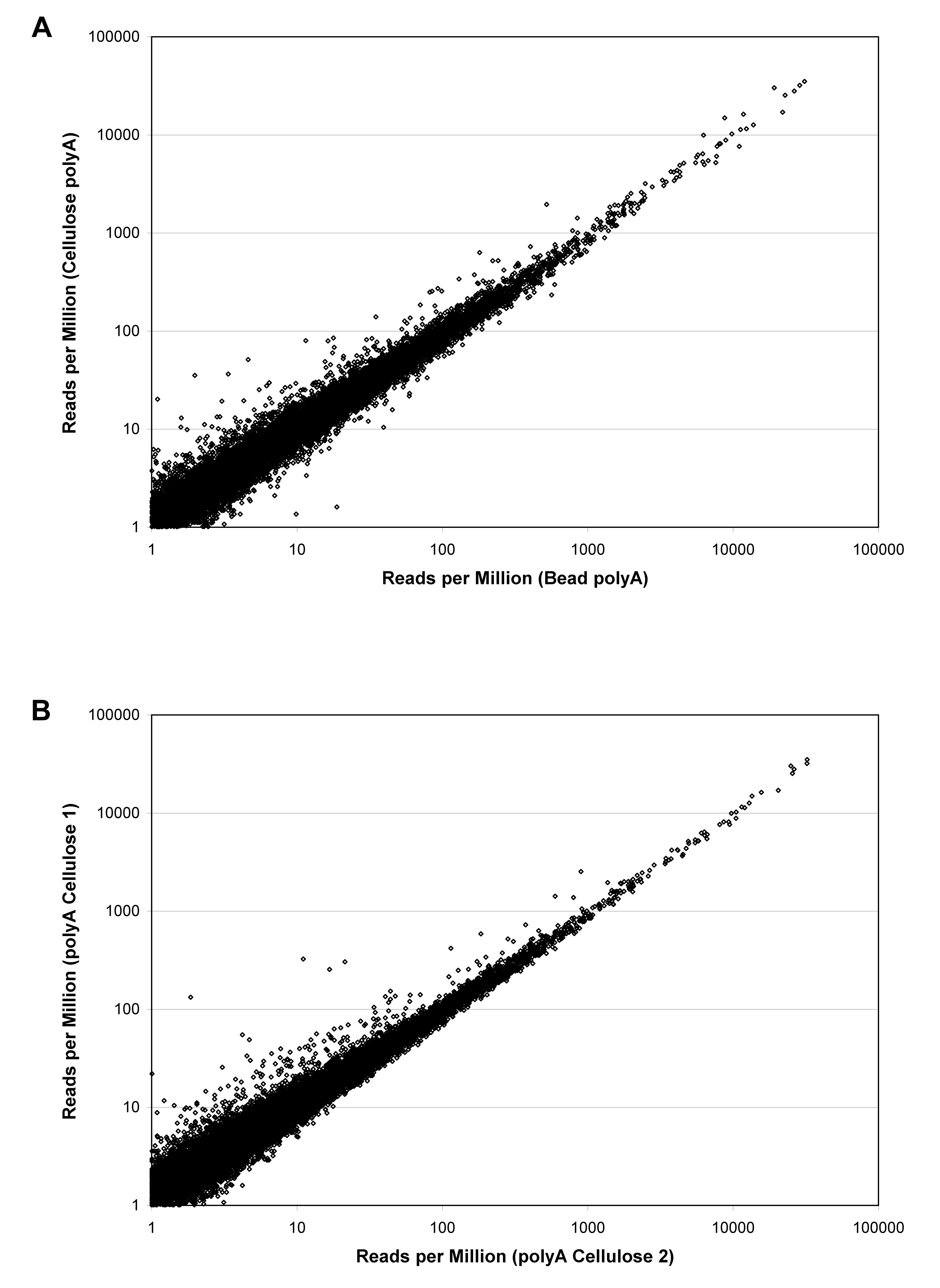

Supplement: Figure S1 — Varying methods of polyA selection. polyA selection of liver RNA was carried out as described in Materials and Methods using either beads or cellulose. Expression levels for once-selected RNA are compared for beads and cellulose (A). Additionally, a fraction of the polyA RNA selected once with cellulose was selected again with cellulose to generate highly selected polyA RNA. The expression differences between once and twice selected RNA are shown (B). (TIF) [file pone.0019287.s001.tif]

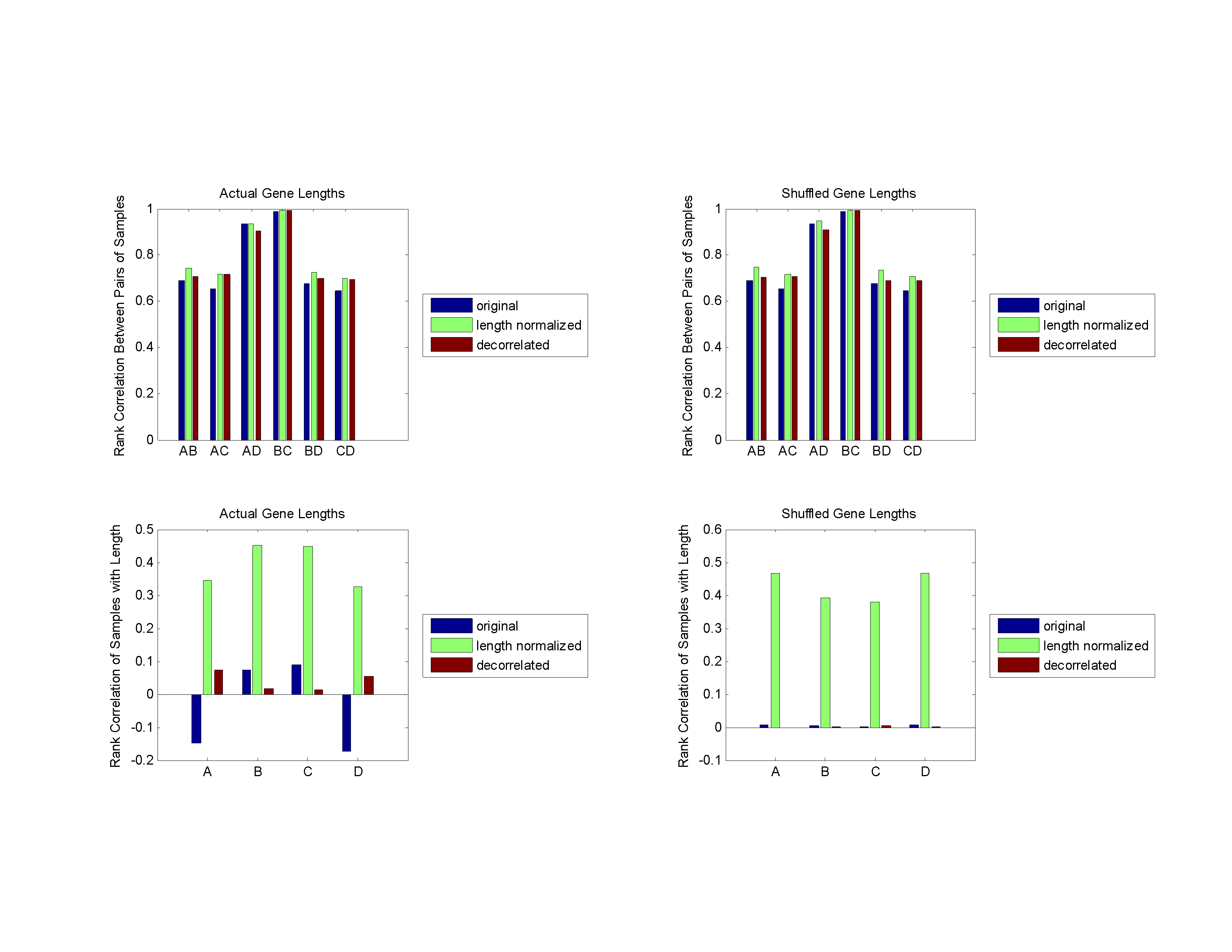

Supplement: Figure S2 — Artifactual correlations induced by length corrections. Four different liver RNA samples were compared pair-wise for correlations with varying methods of correcting for transcript length. (TIF) [file pone.0019287.s002.tif]

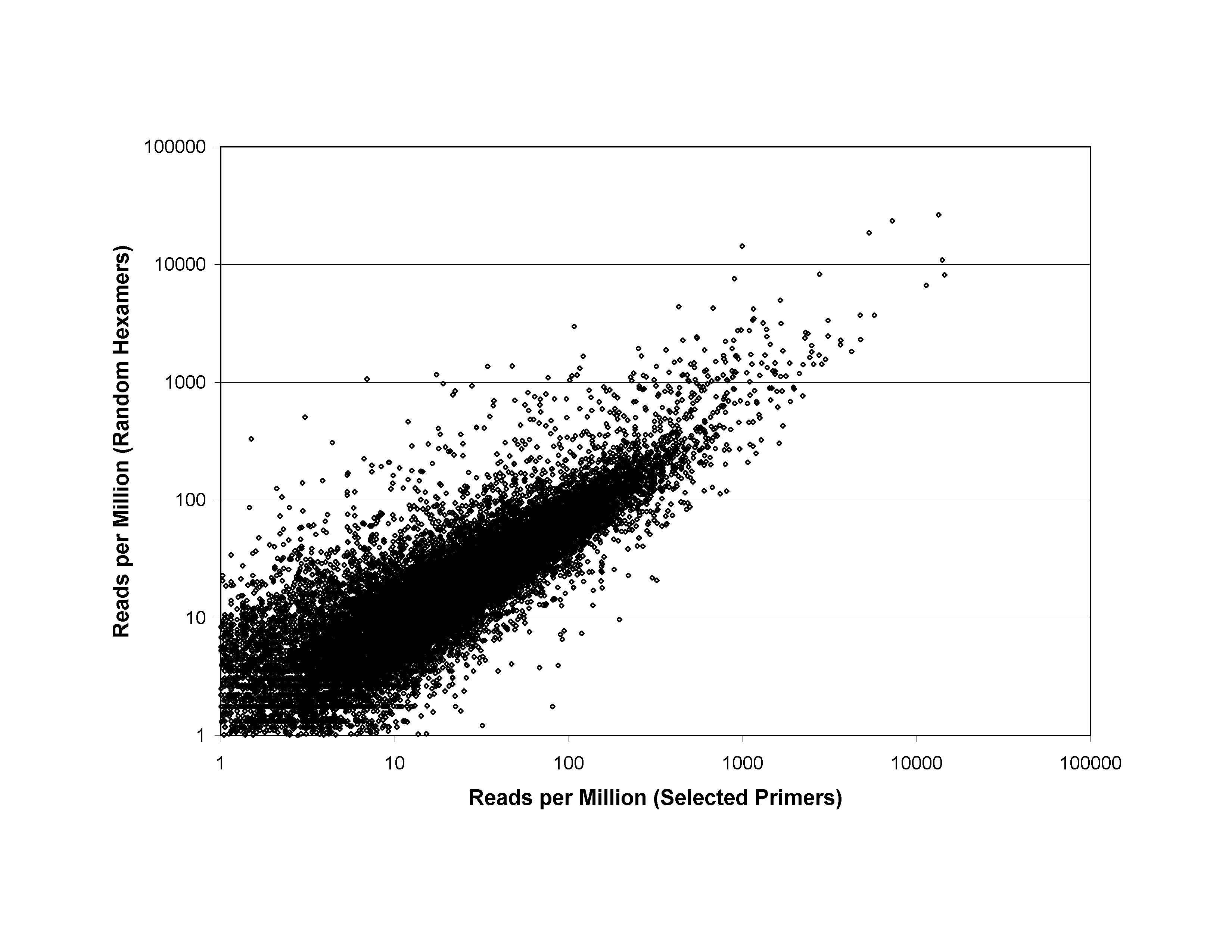

Supplement: Figure S3 — Selected versus random hexamer priming in HL60 total RNA. cDNA was synthesized from HL60 Total RNA using either random hexamers or hexamers selected to avoid cDNA synthesis from ribosomal RNA. Expression levels for transcripts with more than 5 RPM in either sample are shown on a log-log plot. (TIF) [file pone.0019287.s003.tif]

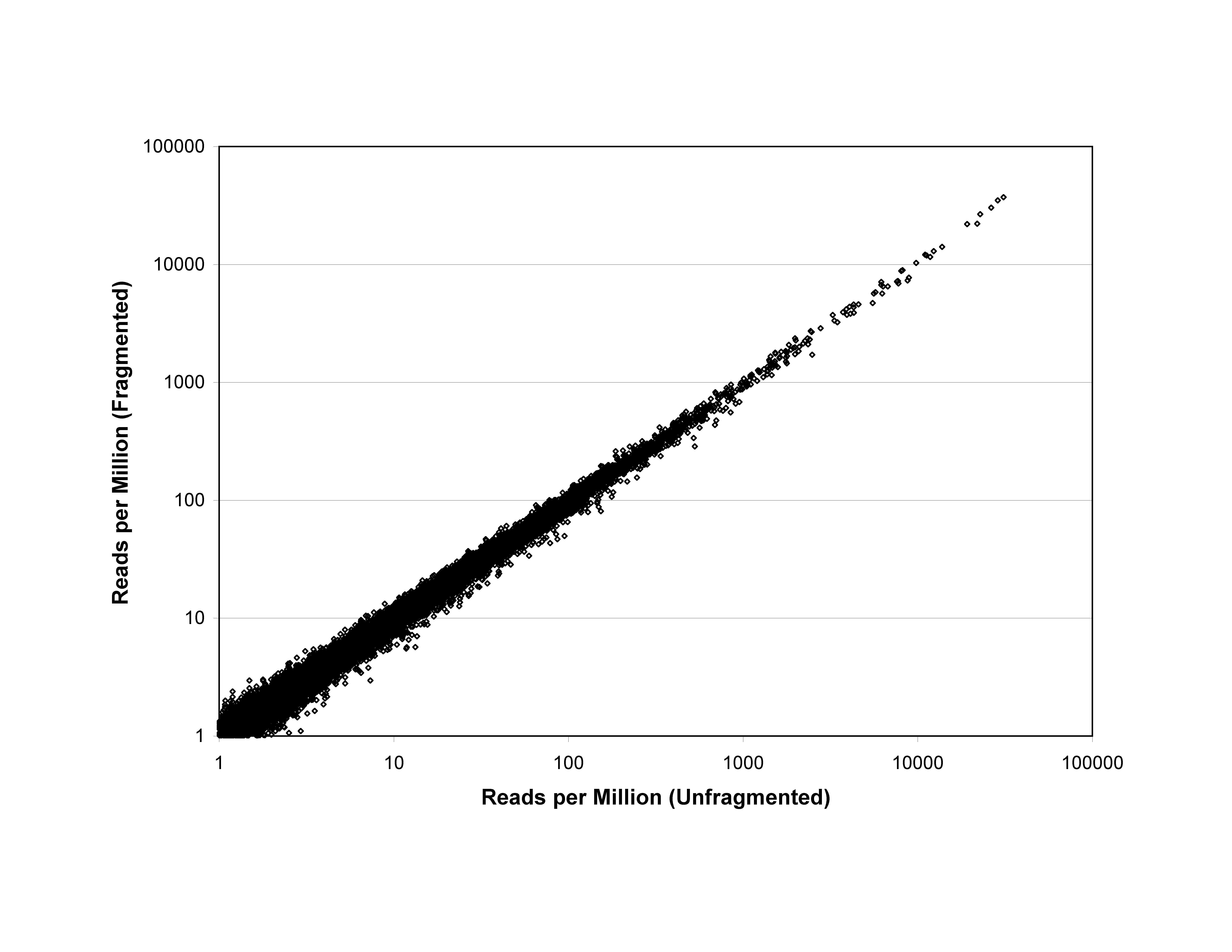

Supplement: Figure S4 — Fragmented versus unfragmented liver polyA. polyA RNA was prepared from liver. Prior to cDNA synthesis, RNA was fragmented by heating to 95° in MgCl2. RNA was then prepared identically and sequenced. RPM for fragmented and unfragmented RNA is shown on a log-log plot. (TIF) [file pone.0019287.s004.tif]

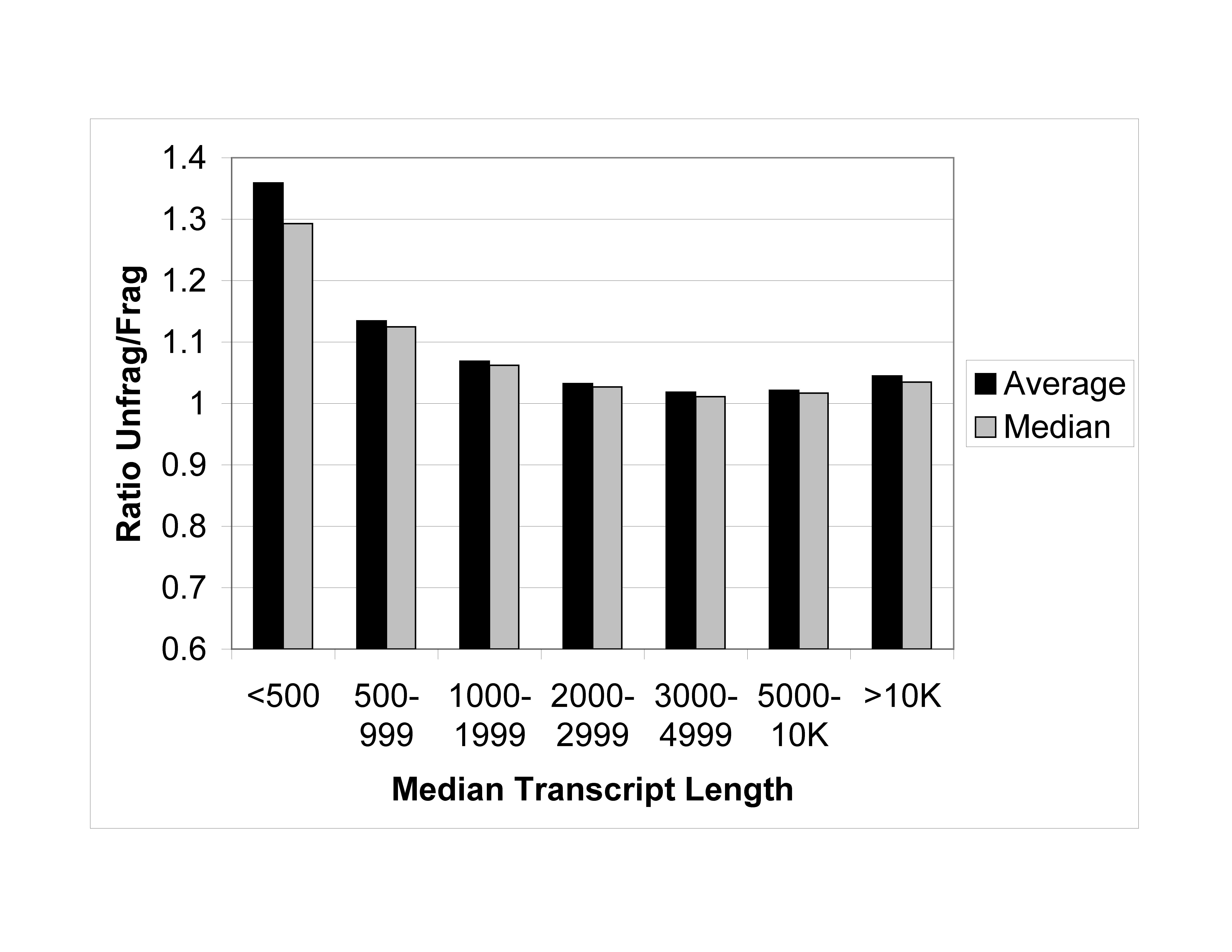

Supplement: Figure S5 — Ratio of expression for unfragmented/fragmented liver polyA RNA. Liver RNA was fragmented after polyA selection as described in Figure S1. The ratio of expression was determined for all transcripts with greater than 50 RPM in either sample. Transcripts were then binned based on the UCSC median transcript length and average and median ratios of expression determined for each bin. (TIF) [file pone.0019287.s005.tif]

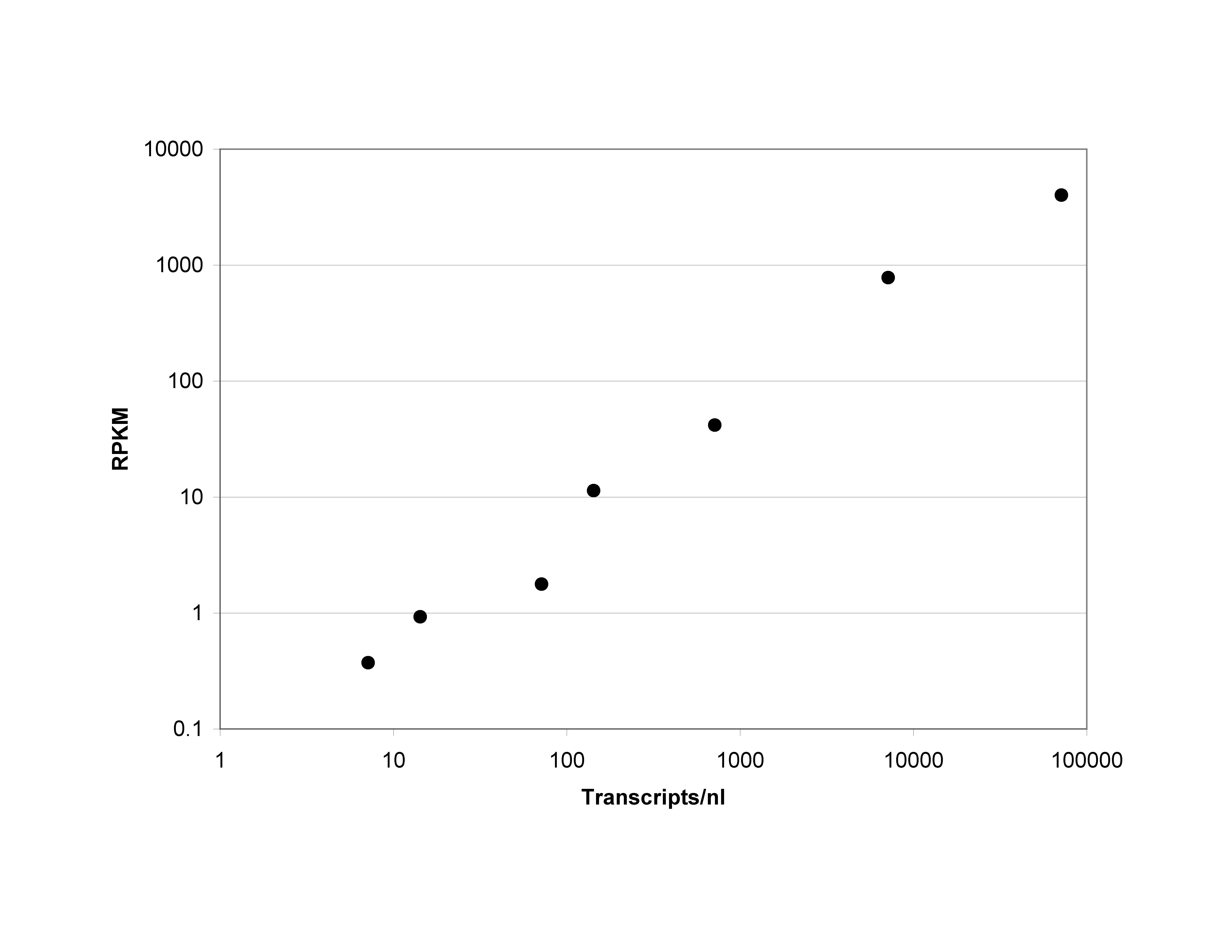

Supplement: Figure S6 — Spike-in expression. Spike-in RNAs were provided by Dr. Brian Willi in Dr. Barbara Wold's laboratory. These were added to a liver RNA sample and prepared as described above. Because the absolute number of molecules in the sample was known, the counts were adjusted for the known lengths and then plotted versus the known spike-in concentration. The number of transcripts per nanoliter of spike is shown on the horizontal axis and RPKM on the vertical axis. The single point below the diagonal is AGP which is only 325 nt long and represented by 3 reads and thus the least precise measurement among the spiked RNAs, reinforcing the issues with converting RPM to RPKM. Although two transcripts have lower RPKM than AGP, they are, in fact represented by more reads (19 and 7, respectively) because they are much longer (9786 and 1451 nt). (TIF) [file pone.0019287.s006.tif]
