# Supplementary material for: Protocol Dependence of Sequencing-Based Gene Expression Measurements
Source: PLoS One. 2011 May 6;6(5):e19287. doi: 10.1371/journal.pone.0019287 (PMC3089619; doi:10.1371/journal.pone.0019287)
Supplement: Text S1 — Additional details on how sequence reads were aligned and counted, how normalization and correlations of data were carried out, and how spikes and expression differences were analyzed is provided. (DOC) [file pone.0019287.s008.doc]

**Supporting Text S1**

***Mapping single-molecule (SMS) reads to the genome and annotated transcriptome.***

Filtered SMS reads were trimmed for leading T homopolymers and were filtered for reads with a minimal length of 25 bases for the whole genome alignments or 24 bases for alignment to the transcriptome after trimming using a suite of Helicos tools available at: <http://open.helicosbio.com/mwiki/index.php/Releases> and described at: <http://open.helicosbio.com/helisphere_user_guide/2009-R1/index.html>. Alignments were conducted with indexDPgenomic software freely available on the Helicos website (<http://open.helicosbio.com/mwiki/index.php/Releases>). The aligner maximizes the aligned yield of SMS reads due to the ability to align reads in which the predominant error is represented by deletions common in single molecule sequencing [1]. For the genomic alignments, reads were aligned to the NCBIv36 version of the genome supplemented with the complete ribosomal repeat unit (Gen. Bank. Accession U13369.1) using the Helicos BASIC analysis pipeline (<http://open.helicosbio.com/helisphere_user_guide/2009-R1/index.html>). Reads with a minimal normalized alignment score (see below) of 4.5 and above were allowed. When determining where a sequence read aligns, one can choose only unique alignments or choose to partition the reads based on multiple possible alignment locations [2]. Such a partitioning can be done in many ways which generally yield reasonable results as long as performed consistently. Inclusion of non-uniquely aligning reads is required if transcripts with repeated regions are to be accurately assessed. In these analyses, reads were assigned on a fractional basis to all equivalently aligning positions though alignments using only uniquely aligning reads or assigning reads based on expression level [3] is possible and yields similar overall results.

For the transcriptome alignments, reads were aligned to the collapsed annotations of known genes derived from the UCSC Genes track on the UCSC browser [4] on both the forward and reverse strands using the Helicos DGE analysis pipeline (<http://open.helicosbio.com/helisphere_user_guide/2009-R1/index.html>). For each annotation, the number of aligned reads with a minscore of 4.3 was counted as previously described [3] with the exception that reads mapping to more then one location with the same score were evenly split among the locations.

The normalized score was defined as following;

Score=(#matches*5-#mismatches*4)/length_read

For example, in the following alignment:

Tag Sequence CCTCCGTGTTGTTCCAGCC-CAGTGCTCGCAGG

Ref Sequence C-TCCGTGTTGTTCCAGCCACAGTGCTCGCAGG

Length of alignment block: 33

Length of tag sequence: 32

Number of matches: 31

Number of errors: 2

Score: (31*5) - (2*4) = 155 - 8 = 147

Normalized score = 147/32 = 4.59375

***Genomic bin analysis and comparison with annotations.***

Reads uniquely mapping to the human genome were pruned for the ones derived from the complete ribosomal repeat unit, mitochondrial genome and the ribosomal repeats as defined by the RepeatMasker track on the UCSC browser [4]. The remaining reads were compared with exons of known genes as defined by the UCSC Genes track on the UCSC browser [4] to estimate the fraction of reads that overlap exons.

To perform an unbiased profiling of transcripts (both annotated and unannotated), the genome sequence was split in 100bp bins and the number of reads (defined by the left coordinate) was counted in each bin for each sample and normalized to 10 million reads. The bins that had at least 9.5 reads per 10M where used to transcripts differentially (at least 3 fold) expressed between different tissues. Bins that had 0 reads were assigned the value of 0.5 reads per 10M.

**Correlations and length corrections**

For expression data, Spearman correlations were performed though similar results are obtained when log transformed data are analyzed using Pearson correlations. Generally, transcripts from the samples being compared were only included if at least one of the samples had >5 RPM. Removal of very low expressing transcripts avoids over-representing transcripts that are not expressed in a given tissue. Inclusion of thousands of non-expressing transcripts can artificially improve a correlation and make informative comparisons across experiments more difficult. This is exemplified with the Illumina data presented elsewhere [5]. The authors report a correlation of 0.866 for two libraries prepared from the same RNA. If we align their reads to our standard transcriptome and compare all 28,808 genes, we observe a correlation of 0.82. However, if we eliminate the 12,938 transcripts with fewer than 5 RPM in both libraries, the correlation drops to only 0.48. Thus, over-inclusion of poorly expressed genes in a correlation can lead to deceptively high values. Similarly, comparison of correlations should be carried out for samples with a similar number of reads whenever possible. Care must be taken not to mistake a low correlation due to fewer reads with a low correlation due to poor reproducibility.

Generally, correction for transcript length was not carried out. Except for the spike experiments, all of our analyses were comparative rather than absolute so normalization was not necessary. In fact, length normalization can both introduce issues and obscure the degree of correlation. Short transcripts are typically the most difficult to detect due to fewer cDNA priming sites and hence they are more susceptible to artifacts during processing. However, they are also the ones that are subjected to the largest correction factor. This eliminates the simple Poisson connection between apparent expression level and precision and these transcripts will appear off the diagonal much more often than longer transcripts with similar RKPM. Furthermore, correcting for length is not straightforward as most annotated transcripts have multiple lengths, sometimes varying considerably. Choosing one length for a transcript can be somewhat arbitrary and based on limited experimental information.

The RPKM length normalization [6] can artificially inflate correlation coefficients. Division by length is equivalent to multiplying each gene expression profile by the same reciprocal-length vector, which can make them more similar, increasing their correlation coefficients. To explore this issue we looked at the effect of length normalization on pairwise rank (Spearman) correlations among four samples (liver polyA from Scientist 1 and Scientist 2, liver RiboMinus, and liver Total RNA). For pairwise correlations that were not already saturated (>0.99), division by the median transcript length  increased the average correlation by 0.04 (Figure S2, upper left). Interestingly, the increase in correlation is similar (.05) even if the gene lengths are randomized (upper right), demonstrating that the effect is non-specific. We also looked directly at the correlation of gene expression and reciprocal length, and found that the unnormalized data showed small correlations with length, both positive and negative, ranging between -0.2 and 0.1 (lower left, blue). The length-normalized data, by contrast, showed significant correlations with length between 0.33 and 0.45 (lower left, green). Randomizing the length assignments eliminated the length correlations in the untransformed data but left similar magnitude correlations in the transformed data (lower right), suggesting that the length correlations in the original data may be real, but that length normalization induces artifactual length correlations.

We explored an alternative length normalization process we call "decorrelation", in which we divide the expression vector element-wise by the length vector raised to a power that minimizes the correlation of the result with length. This is equivalent to removal of an additive length factor in log space. This procedure produces transformed data that show less correlation with length than the original data (lower left, blue vs. red), and significantly less length correlation than the length-normalized (RPKM) data. The decorrelated data show a slight increase in pairwise correlation over untransformed data (mean=0.02), suggesting some overnormalization, but the effect is smaller than with length normalization (upper right, red). When the lengths are randomized, decorrelation again shows a mean artifactual correlation increase of .02 vs. .05 for length normalization. Surprisingly, the decorrelation procedure does not appear to induce any artifactual correlation when applied to Pearson correlations in log-space (not shown), while length correlation is completely eliminated. We conclude that if length normalization is desired, correlation removal appears to be a less biased approach than RPKM-like length normalization. However, the fact that both positive and negative correlations with length are observed in untransformed data suggests that this issue needs to be approached with care; it may be advisable to first understand the source of the length correlations – whether driven by underlying biology, or sample preparation effects – before removing them from the data. In any case, when correlation coefficients are used to compare across sample prep methods or platforms, it is critical not to apply length normalization selectively, since that will inflate some correlations and not others.  When comparing data across laboratories, it is important to know whether data has been “length corrected” or not, how the correction was done, and the potential impact this has on the relative correlation values.

**Spikes**

One special case where the use of length corrected data is appropriate is with spiked-in RNAs of known length and mass. While these are still subject to the issue of length not being a perfect predictor of how many reads will be present, the known lengths and known masses of the spike-ins allow absolute comparisons to be made. For some experiments, *in vitro* transcribed RNAs were added to the complete RNA sample prior to cDNA synthesis. These spike-ins were kindly provided by Dr. Brian Willi in Dr. Barbara Wold’s laboratory. These spikes have been used previously [6] and shown to perform well. The spike mixture was added to a polyA RNA sample and carried through the entire process within the bulk RNA sample then aligned to the known sequences along with the rest of the RNA from the liver RNA. Counts ranged from 3 for the least common RNA (AGP) to 29,836 for the most common, OBF5, in a background of 5,201,898 non-rRNA, non-mitochondrial reads. The known transcript lengths were used to normalize for RPKM. The resultant log(RPKM) was correlated with log(mass RNA) with R>0.99 (Figure S6). One of the lambda RNAs, though performing well in the standard protocol, yielded higher than expected RPKM for fragmented samples for unknown reasons.

**Differential Expression in HL60**

The human cancer cell line HL-60 was purchased from the American Type Culture Collection (ATCC; CCL-240). All cell manipulations were carried out in a laminar flow biosafety hood. Cultures were established in ATCC recommended media (Iscove’s Modified Dulbecco’s Media (MediaTech 10-016-CV) containing 20% fetal bovine serum (FBS; Gibco 10082, lot # 1412312) and 1% penn/strep/glutamine (PSG; MediaTech 30-009-CI) in Nunc T175 flasks and incubated at 37ºC in humidified 5% CO2 incubators. The cells were passaged twice before adapting to RPMI 1640 media (MediaTech 10-040-CV) and only 10% FBS and 1% PSG. Adaptation occurred by culturing the cells in RPMI

1640/20% FBS/1% PSG followed by 15% FBS and then finally 10% FBS over the course of three passages. Cells were passaged every 48 to 72 hours. Whenever HL-60 cultures were passaged, they were counted and split into 30 mL fresh media to a density of 2.0 x 10^5^ cells/mL in T175 Nunc flasks. Cultures were split regularly to maintain log phase growth. HL-60 cells were cultured in the presence of an agent that initiates differentiation, all trans retinoic acid (tRet) by MIR Preclinical Services (Ann Arbor, MI). Total RNA was isolated using RNeasy Maxi columns (Qiagen) after the cultures were treated for 5 days.

t-Ret produces substantial changes in expression. If one examines the transcripts that are expressed at >50 RPM in either control or t-Ret-induced samples, there are 25 transcripts that are at least 10x downregulated in the total RNA. Of these, all are downregulated in both the polyA and DGE samples with 14 also downregulated at least 10x in each sample. In the total RNA sample, 52 transcripts are upregulated at least 10x, all of which are upregulated in both the polyA and DGE samples. 49 and 50 of the upregulated transcripts, respectively, are also upregulated at least 10x in the polyA and DGE samples.

**References**

1. Giladi E, Healy J, Myers G, Hart C, Kapranov P, et al. (2010) Error tolerant indexing and alignment of short reads with covering template families. J Comput Biol 17: 1279-1293.

2. Taub M, Lipson D, Speed TP (2010) Methods for allocating ambiguous short-reads. Communications in Information and Systems 10: 69-82.

3. Lipson D, Raz T, Kieu A, Jones DR, Giladi E, et al. (2009) Quantification of the yeast transcriptome by single-molecule sequencing. Nat Biotechnol 27: 652-658.

4. Kent WJ, Sugnet CW, Furey TS, Roskin KM, Pringle TH, et al. (2002) The human genome browser at UCSC. Genome Res 12: 996-1006.

5. Mamanova L, Andrews RM, James KD, Sheridan EM, Ellis PD, et al. (2010) FRT-seq: amplification-free, strand-specific transcriptome sequencing. Nat Methods 7: 130-132.

6. Mortazavi A, Williams BA, McCue K, Schaeffer L, Wold B (2008) Mapping and quantifying mammalian transcriptomes by RNA-Seq. Nat Methods 5: 621-628.
